# A Malposed Tooth

**Published:** 1900-02-15

**Authors:** C. D. Peck

**Affiliations:** Sandusky, Ohio


### CLINICAL CASES.

Presented at Ohio State Dental Society, December, 1899.

### A MALPOSED TOOTH.

BY C. D. PECK, D.D.S., SANDUSKY, OHIO.

About three months ago a case was presented where the first and second molars were missing; apparently a root had been left which was below the gum. On investigation, I found a third molar tooth lying in a horizontal position, with the grinding surface toward the rear, which had never been erupted. I succeeded in removing the tooth. It extended through the floor of the antrum. I could pass an instrument into the antrum. I have been able by antiseptic treatment to nearly make a cure.

The patient lived eight miles in the country, so could not visit me very frequently. After that tooth had been treated for four weeks, there was an opening in the process. I saw him again a few days ago and it was just about closed.

